# Saving the Teeth

**Published:** 1894-05

**Authors:** W. Irving Thayer


ARTICLE II.

### SAVING THE TEETH.

BY W. IRVING THAYER, D. D. S., M. D.

Before we consider a system calculated to increase the resistance of teeth to decay, it will be more profitable to the reader to explain the most approved methods to be employed to save and prevent the decay of the natural teeth. Apart from their structure, an important point to be carefully guarded against is the prevention of any accumulation of foreign substances upon or around the teeth.

Any acid, like vinegar, will dissolve the lime of the teeth and destroy their solidity. Vegetable and animal food remaining between the teeth will, after a while, become an acid of more or less strength which will prove most injurious to the teeth.

Teeth should be cleaned as much as four times a day, after each meal and just before going to bed. More people lose their teeth from this neglect than from any other cause.

The writer has frequently seen children and others eating limes and lemons with as much pleasure as one would have in eating a very sweet Florida orange. It would be difficult to induce such persons to hold in their mouths diluted sulphuric or nitric acid, yet they will take very strong citric acid found in fruit with apparent relish. There are some sorts of apples that have a large amount of acetic acid which is quite destructive to the teeth. One who loves apples can indulge in such fruit if he will thoroughly clean his teeth immediately after eating. Mothers should personally see that their children clean their teeth thoroughly and regularly. Children do not realize the great importance of such a procedure, and are very liable to shirk their duty in this matter. The rule among the little folks is to slight such an operation.

There are but a very few persons who know how to easily and perfectly clean their teeth. Ten persons out of twelve swing the brush across the visible diameter of the teeth, or at right angles with their perpendicular plane. If this is done with a stiff brush for a series of months the operator will surely cut furrows on the lip or cheek side of the teeth and do them a great injury. No matter how hard one's teeth may be, this result will sooner or later follow such a method of using the toothbrush.

The proper way to brush and clean the teeth is to brush from the gums downward, for the upper teeth, and from the gums upward, for the inferior or lower teeth. By this method the bristles go between the teeth, touching their approximating surfaces, as well as cleaning the front and sides of the teeth.

It is not less important to brush downward on the palatine-roof surface of the upper teeth, and upward on the lingual-tongue side of the lower teeth, that is to say, brush the inside of the teeth more carefully, if anything, than the outside. Do not brush and clean the teeth for the sole purpose of making them look bright, clean and pretty, but to keep foreign substances from producing caries, or, plainer still, eating holes into the teeth and exposing a highly sensitive tissue, the pulp or nerve. Clean the grinding surfaces of the teeth with the same interested care. If economy is wealth, this is one plain road to the condition. Nay, more, while one saves dental bills, he saves, what is infinitely of more value, his teeth. It is not contended that there is no necessity to consult the family dentist. This should be done much more frequently than it is. A small cavity arrested in decay is much better than to have a large decayed spot to fill, or the ultimate loss of the tooth. But the highest art in dentistry is to prevent the formation of cavities, and not to fill them.

Women in general are supposed to be greatly interested in the preservation of their teeth, and it may be somewhat painful to them for me to inform them that they do not generally possess as strong and dense teeth as do the male sex. That is the rule, the exceptions are rare. Hence the importance of cleanliness on their part is more important. Then, again, mothers will do well to remember that the bones and teeth of their children are not as firm and compact as are these tissues in adults. There has not been deposited amidst the soft-solids of these textures as much of the carbonate and phosphate of lime as we will find as they grow older. Hence the value of preventing acid formotion.

The approximating surfaces, or the sides of the teeth that touch each other, are very important places to be guarded from foreign accumulations. Do not use pins, needles, metallic toothpicks or any hard substance, or anything as yielding as a piece of hard wood, like a silver of beech, oak or walnut, but a thin goose quill, soft basswood picks, or broomcorn. Endeavor to cause the toothpick to rub against the side of each tooth so that anything adhering to the perpendicular walls of the teeth may be removed. Floss or any soft silk thread may also be used to great advantage.—*Ladies' Home Journal.*

